# A Feasibility Study on Extension of Measurement Distance in Vision Sensor Using Super-Resolution for Dynamic Response Measurement

**DOI:** 10.3390/s23208496

**Published:** 2023-10-16

**Authors:** Dooyong Cho, Junho Gong

**Affiliations:** 1 Department of Convergence System Engineering, Chungnam National University, Daejeon 34134, Republic of Korea; dooyongcho@cnu.ac.kr; 2 Department of Future & Smart Construction Research, Korea Institute of Civil Engineering and Building Technology, Goyang-si 10223, Republic of Korea

**Keywords:** dynamic displacement measurement, environmental limitation, feasibility study, feature point tracking, image sensors, super-resolution

## Abstract

The current civil infrastructure conditions can be assessed through the measurement of displacement using conventional contact-type sensors. To address the disadvantages of traditional sensors, vision-based sensor measurement systems have been derived in numerous studies and proven as an alternative to traditional sensors. Despite the benefits of the vision sensor, it is well known that the accuracy of the vision-based displacement measurement is largely dependent on the camera extrinsic or intrinsic parameters. In this study, the feasibility study of a deep learning-based single image super-resolution (SISR) technique in a vision-based sensor system is conducted to alleviate the low spatial resolution of image frames at long measurement distance ranges. Additionally, its robustness is evaluated using shaking table tests. As a result, it is confirmed that the SISR can reconstruct definite images of natural targets resulting in an extension of the measurement distance range. Additionally, it is determined that the SISR mitigates displacement measurement error in the vision sensor-based measurement system. Based on this fundamental study of SISR in the feature point-based measurement system, further analysis such as modal analysis, damage detection, and so forth should be continued in order to explore the functionality of SR images by applying low-resolution displacement measurement footage.

## 1. Introduction

Infrastructures have been constructed to satisfy and improve both social needs and the quality of life for the public. Whereas they maintain convenience, various types of loads and environmental impacts have decreased their service life and functionality and have further led to structural failures. Many developed countries have focused on the present condition of civil engineering structures and have reported the request for a reasonable maintenance regime including prioritization of backlogs of repair needs to prevent the deterioration of civil engineering structures [1,2]. To accomplish the prolongation of structures’ serviceability, it is inevitable to investigate their structural health condition [3,4,5].

The current status of infrastructures should be periodically examined through the measurement of various structural responses in order for preventative maintenance. Amongst the physical quantities, displacement data are an imperative consideration in broad engineering areas such as deflection measurement [6], load estimation [7,8], damage detection [9], model updating [10], modal analysis [11], and so forth. To obtain the structural responses, conventional sensors such as linear variable differential transformers (LVDTs), accelerometers, and/or strain gauges are used and installed during the examination or monitoring of structures. Whereas these sensors have been proven to provide reliable measurement data, they often are limited in spatial resolution or demand installation of dense sensor arrays [9,12,13]. Such conventional sensors, for instance, the LVDTs, need to be installed at the stationary reference points and to be physically contacted on the surface of measurement points [13,14]. Such installation of traditional sensors can be time-consuming, laborious, and expensive to operate or vulnerable to health and safety issues. Furthermore, direct access to the structures to set the sensors could occasionally be difficult or limited owing to low accessibility [4,15].

To address the disadvantages of contact-type sensors, noncontact-type sensors (e.g., Global Positioning Systems (GPSs), Laser Doppler Vibrometers (LVDs), and radar interferometry systems) are considered as the traditional sensors [16,17,18]. The GPS sensors have gained attention owing to their convenience of installation but have the limitation of measurement accuracy (errors between 5 and 10 mm) [17,19]. The LVDs perform general measurement accuracy in a short measurement range. Even though the high-intensity laser beam allows for an extension of the distance, this causes health and safety issues [17,18]. The interferometric radar can measure structural responses with a good resolution, but reflecting surfaces must be placed on the structure, which makes it cumbersome and time-consuming [20].

Due to the development of vision sensors, the cost range of high-performance digital cameras, which have high shutter speeds and high resolution, has been reduced, making them more affordable. With the advancement of efficient computer vision algorithms, vision sensors have become improved surrogates for conventional contact-based sensors. During the last decade, a large number of studies have been assessed to obtain quantitative information from a video [21]. Compared to the conventional sensors, the vision-based monitoring methods have distinctive aspects such as convenient instrumentation, simple installation, remote measurement, and capacity for multi-point measurement using a single sensor [3,4,5,7,13].

Despite the merits of the vision sensor, monitoring structures remotely using a single camera is challenging in terms of engineering applications [3,5,19,22]. The following three concerns pose limitations in its practical applications: (1) Most vision-based displacement sensors require a predesigned high-contrast target panel, which needs to access the structure to install the target panel. (2) It is well known that the accuracy of the vision-based displacement measurement is largely dependent on the image quality, which is often difficult to guarantee in outdoor field environmental conditions such as illumination variation and optical turbulence. (3) The accuracy of this measurement system is sensitive to the operation distance and camera intrinsic parameters.

The vision-based measurement systems are susceptible to certain environmental limitations such as illumination fluctuation, measurement distance, or optical turbulence in the field in hot weather [23]. Among them, the measurement distance and range are crucial factors that engage not only operational capacity but also the measurement performance of the vision sensor. The spatial resolution in digital images depends on the spatial density of the digital image. The sampling interval, which is the distance between each pixel, increases as the field of view becomes wider. To obtain definite images over a long distance, a long focal length lens or denser imaging sensor arrays are required [24].

In this connection, it would be a logical idea to simply upgrade the optical imaging system. Such an approach is not efficient in terms of cost due to the regular substitution of the hardware. Furthermore, it is applicable for taking new high-resolution images but is not used to enhance the resolution of existing low-resolution images. As the computer vision area has progressed, image resolution enhancement based on signal processing has received increasing attention owing to its flexibility and economic feasibility [25]. Super-resolution is the task of reconstructing the higher spatial resolution of a given low-resolution image. With super-resolution techniques and deep-learning architectures, images with a resolution beyond the limit of imaging systems can contribute to consecutive image processing analysis such as semantic segmentation [26,27], detection [28,29,30], and recognition [31,32]. Recently, the upscaled images obtained by using the super-resolution techniques have been implemented to analyze structural deformations by using stitched multiple image frames of the beam structure [33] and to improve displacement measurement accuracy using surveillance video cameras [34]. There are a couple of studies conducted to define the capabilities of super-resolution techniques in civil engineering areas, whilst their feasibility for measuring structural responses has not yet been broadly investigated.

The objective of this study is to systematically explore the feasibility of a super-resolution model in a vision sensor-based remote sensor to enhance measurement performance and extend operation capacity. Additionally, the robustness of the vision sensor with super-resolution images is experimentally investigated under various measurement distances.

## 2. Methodology of Dynamic Displacement Measurement Using Single Image Super-Resolution

The SISR methods are normally interested in restoration and refinement applications in computer vision areas. Although most researchers focus on improving the SR performance of the model, the implementation of SISR in vision sensor-based measurement systems is rarely conducted for extending measurement distance. This section considers brief explanations of a feature point-based displacement measurement system and a methodology of its measurement performance depending on the increase in measurement ranges. In addition, the SISR method implemented in this study is explored and its feasibility and robustness study are explained.

### 2.1. Feature Point-Based Measurement System

To obtain the dynamic response of structures from image sequences, there are various tracking approaches in terms of computer vision such as model-based, region-based, active contour, and feature-based methods. Amongst these methods, the template matching technique-based measurement schemes that use artificial targets as reference markers have traditionally found outstanding performances in former studies [3,4,13]. It is also found that tracking motion trajectories using key points extracted from objects within images shows good displacement measurement performances [5]. However, it requires a fundamental task to define which features are good to detect, or how to track them as well [35].

The interest points are normally detected in the form of corners, blobs, edges, junctions, lines, and so forth. Researchers suggested several methods such as tracking corner points, matching image patches with a high spatial frequency content or image regions where the mix of second-order derivatives is high enough to overcome the aperture problem [36]. Additionally, highly discriminative or salient features are required for the achievement of reliable tracking objects [5]. They should be invariant to illuminance fluctuation, scale, and geometry. To accomplish feature approach displacement measurement, two sets of feature detectors, i.e., the Shi-Tomasi corner detector [36] and the KAZE feature detector [37], which are incorporated with the Kanade–Lucas–Tomasi feature tracker algorithm [38], are derived into the vision sensor system.

**Figure 1:**
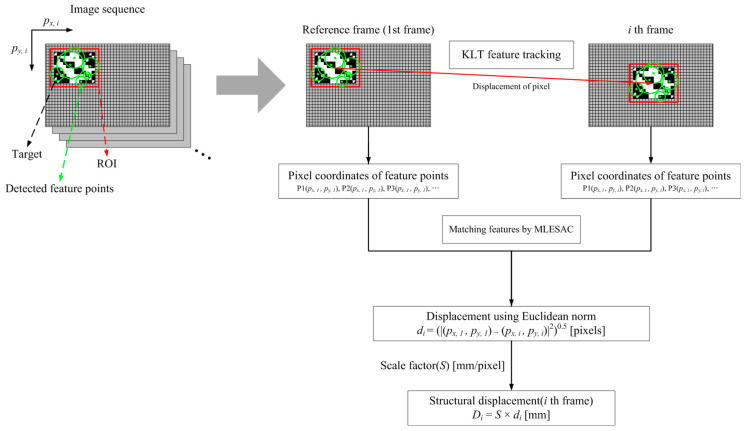
Flowchart of structural displacement measurement based on feature points.

Figure 1 indicates a framework of the measurement of dynamic response using feature points. To begin with, the first frame of an image sequence is used for the reference image to select regions of interest (ROIs) for the detection of key points. The ROIs can be either predesigned markers (high contrast) or natural targets (low contrast) existing on the structure, such as bolts and nuts and so forth. After selecting ROIs, feature points ($P_{n}$) within the ROIs are extracted by either the Shi-Tomasi corner detector [36] or the KAZE feature detector [37] and are registered for identifying the initial position of the subject in pixel coordinates as denoted $P_{n}(p_{x,\;1},p_{y,1})$. The multiple key points $P_{n}(p_{x,\;i},p_{y,i})$ detected in the ith frame are matched with the registered features and tracked by implementing the KLT tracker algorithm [38,39,40]. The Maximum Likelihood Estimation Sample Consensus (MLESAC) method [41] is used to define dominant geometric transformation and to eliminate outliers amongst the matched features for consistent displacement vectors between the reference and ith images. The structural displacement ($D_{i}$) using a vision system is computed by a simple product of the distance vector of the inlier estimated by the Euclidean norm in pixels $d_{i}$ and a scale factor S [42] determined by a correlation of the camera’s intrinsic and extrinsic parameters.

Although the scale factor can be simply obtained from the physical properties of the camera’s parameters, the perpendicularity of the camera’s optical axis to the object surface is mandatory. This prerequisite consequently requires that all measuring points on the object are in the same depth of fields [15,43,44]. The misalignment of the optical axis would lead to difficulties in the implementation of the vision measurement system because a small number of misaligned angles can be imperceptible throughout the experiment setups, specifically as the measurement distance is relatively long. Likewise, in practice, it would be occasionally inevitable to tilt the optical axis to capture structural behaviors.

The measurement errors caused by the nonperpendicularity of the optical axis were evaluated to increase the measurement accuracy of the vision system. Numerical studies of measurement errors between the scale factors depending upon the camera’s perpendicularity were carried out [15]. They found that the measurement errors were estimated to be 0.9% and 1.1% for perpendicular and not perpendicular to the optical axis at a tilt angle of 30°, respectively. Thus, the errors from the small tilt angle could be negligible and acceptable in practical implementations [15]. The camera calibration followed by Zhang’s method [45], which can reduce lens distortion [22,44], can be used to minimize measurement errors, but it was not accomplished in this study.

### 2.2. Indoor Experimental Conditions for Dynamic Displacement Measurements

**Figure 2:**
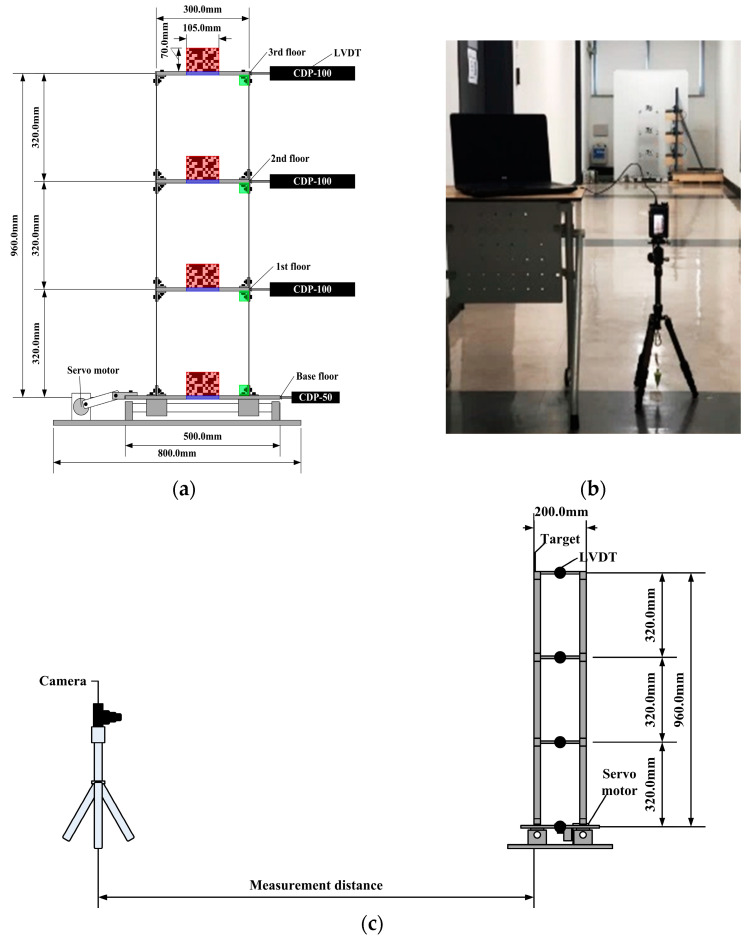
Frame structure and shaking table test configuration. (**a**) Frame model configuration; (**b**) vision sensor setup; (**c**) shaking table test setup (side view).

To evaluate the displacement measurement performance of the vision sensor using single image super-resolution (SISR) as measurement distance increases, a three-story frame structure was prepared for indoor shaking table tests. As described in Figure 2, the aluminum slabs (300 mm × 200 mm) and stainless steel columns (300 mm × 25 mm) are bolt-connected to all the connections. The thickness of the slab and column are 10 mm and 1.5 mm, respectively. The frame model was horizontally excited by reciprocating motions of a shaking table generated by a servo motor (APMC-FAL01AM8K by LS ELECTRIC Co., Ltd., Anyang-si, Republic of Korea). The horizontal displacement amplitude of the base slab was ±14.5 mm, and 9.0 Hz sinusoidal motion was induced to demonstrate the 3rd mode shape.

As reference data, the horizontal displacements of each slab were collected with a sampling rate of 120 Hz by four LVDTs (CDP-50 and CDP-100 by Tokyo Sokki Kenkyujo Co., Ltd., Tokyo, Japan) and a data acquisition device (SCXI-1000 by National Instruments Corp., Austin, TX, USA) during shaking table tests. The LVDTs were installed between each floor of the frame and stationary reference points. Predesigned targets (105 mm × 70 mm) which are high contrast, were fixed on the midpoints of each floor, as shown in red regions in Figure 2a, in order to designate multiple ROIs for extracting and tracking features. Additionally, existing natural targets (bolt shafts and nuts (5 mm and 10 mm in diameter, respectively) and cross-sections of slabs (105 mm × 10 mm)), as indicated in green and blue areas, respectively, were introduced to compare displacement measurement performance depending on the targets’ type.

**Figure 3:**
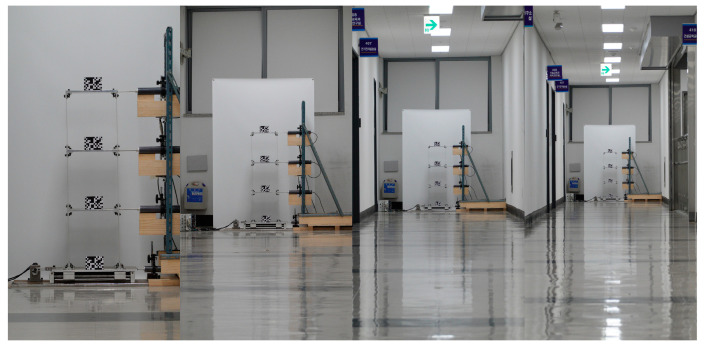
Field of view of the indoor shaking table test. Measurement distance changed from 10 m (**left**) to 40 m (**right**) with 10 m increments.

To capture structural behaviors, a video camera (DSC-RX100M7 Sony Corp., Minato, Tokyo) with a 72 mm focal length, which is 200 mm equivalent to a 35 mm image sensor format, was initially set at a stationary point 10 m away from the shaking table. As illustrated in Figure 2c, the measurement distance, where is the distance between the position of an image sensor and fiducial targets, was surveyed using a total station (GT-501 by Topcon Corp., Tokyo, Japan). The scale factor was 0.95 mm/pixel in this testing configuration. A laptop (P57F Dell Inc., Round Rock, TX, USA) with Imaging Edge Desktop 3.2.01, which is software provided by Sony Corp., was used to align an optical axis of the camera and frame structure and to remotely control recording settings. To obtain sharp image sequences, all the targets were manually focused by observing the magnified view from the abovementioned software. As the harmonic motion was induced, the motion trajectories of the frame structure were digitized into FHD image sequences (1080 × 1920 pixels) for 180 s with a sampling rate of 120 frames per second (FPS). The illuminance was consistent with 450 lux during the whole testing session and the mean ambient temperature of the laboratory in December was 18 degrees Celsius. The displacement measurements were continuously conducted by changing stationary points of the camera sensor with 10 m increments up to 40 m, as shown in Figure 3, and the scale factors corresponding to the measurement distance ranging from 20 m to 40 m were 1.91 mm/pixel, 2.87 mm/pixel, and 3.82 mm/pixel, respectively.

### 2.3. Single Image Super-Resolution in Vision Sensor-Based Measurement System

Various Convolutional Neural Networks (CNN)-based SR networks have successively accomplished remarkable fidelity performance on bicubic downsampling images [46,47,48,49]. In most of them, bicubic operation for downsampling is used to construct training data pairs. Additionally, the downsampled images are fed to the designed network architectures in the test phase [50]. Although these networks have improved fidelity by training to minimize the average error, the generated results using the original image for the test are blurry with significant noise. This mainly is because the bicubic sub-sampled image does not contain the same domain as the original image [50]. Owing to the difference in the domain gap, these models create unsatisfied artifacts when tested with the original image.

**Figure 4:**
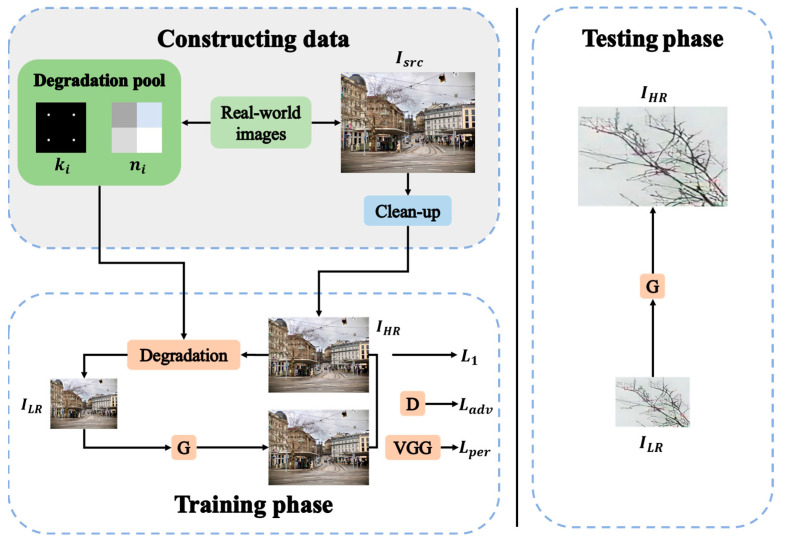
The framework of RealSR method. Adapted with permission from [48]. 2023, Xiaozhong Ji.

Ji et al. [48] found that EDSR [46] and ZSSR [49], for instance, produce unpleasant results in real-world images. The authors also concluded that it is essential to implement a proper degradation technique for real-world super-resolution to generate LR images and maintain the original domain attributes [50]. To deal with this issue, many researchers pay more attention to Generative Adversarial Networks (GANs)-based models by introducing adversarial losses and perceptual losses to improve the visual effects of the artifacts [51,52,53,54]. Ji et al. [50] proposed a novel framework (RealSR) for the SR model to overcome the abovementioned challenges on real-world images. As described in Figure 4, they designed a network following two stages: (1) The realistic blurry LR image is generated by sub-sampling HR images with the estimated degradation. (2) The second stage is the SR training phase using the constructed data.

To overcome the degradation of spatial resolution by implementing the SR, the pre-trained model of RealSR [50] was used for upscaling the image sequences. Because of its outstanding performance, the more realistic super-resolution can be accomplished specifically in the existing features of the structures, which can be directly introduced to the natural targets in vision sensor-based measurement systems with a long measurement distance range. Consequently, the installation of fiducial targets would not be required for the detection of distinctive feature points.

For the validation tests, two sets of image sequences of shaking table tests conducted at 10 m and 20 m were initially used to assess the feasibility of SISR by computing similarity evaluation metrics. Additionally, the displacement measurement error was compared to analyze the displacement measurement performance when the upscaled images were applied. All the image frames were first downscaled with a scale factor of 0.25 and converted to 480 × 270 pixels images. The LR images were reconstructed with a scale factor of 4 through bicubic (BC) upscaling operation and RealSR [50], respectively. The upsampled images (1920 × 1080 pixels) were used for the computation of similarity by evaluation metrics such as Peak Signal-to-Noise Ratio (PSNR), Structural Similarity Index Map (SSIM), and Learned Perceptual Image Patch Similarity (LPIPS) [55]. Furthermore, the image sequences of the displacement measurements at 30 m and 40 m are upsampled with a scale factor of 2. The SR images were equivalent to UHD images (3840 × 2160 pixels) and were used to verify the robustness of SISR in the vision sensor-based measurement system for extending measurement distance.

## 3. Result and Discussion

The dynamic structural responses of frame structure were measured based on LVDTs and estimated by trajectories of feature points within three different types of ROIs. Initially, the robustness of the vision sensor-based measurement system was evaluated by comparing it with the LVDTs’ data as measurement distance increases. In addition, the feasibility of SISR in the vision sensor-based measurement system was assessed, and its robustness for the natural targets is discussed regarding the extension of measurement distance.

### 3.1. Displacement Measurement Performance of Feature Point-Based Measurement System

**Figure 5:**
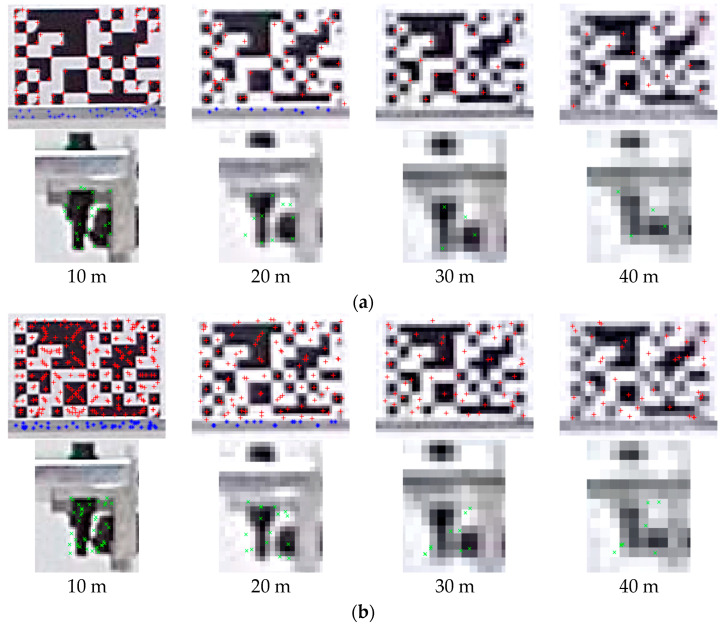
Key points detection results by measurement distance changes. Red +, blue * and green × mean the detected points within the fiducial target, slab cross-section and bolt connection respectively. (**a**) Corner point detection. (**b**) KAZE feature detection.

To evaluate vision sensor measurement performance as the measurement distance increases, three different targets were selected for extracting corner points and KAZE features, respectively. Figure 5 shows the key point detection result comparison of both artificial and natural targets on the 3rd floor according to both feature detection schemes and the measurement distance increments. The image quality of targets is degraded as the measurement distance increases because of the lower pixel density within the allocated ROIs. As shown in Figure 5, the high-contrast target (fiducial target) provides a good reference region for detecting features in both algorithms as the measurement distance increases.

However, as can be seen in the feature detection results of bolt connection in Table 1, fewer quantities of features within the ROIs are detected because the coarser image pixels contribute to obscuring the bolt details. Thus, fewer spatial densities are composed of the images as the measurement distance range increases. Because of this pixel blocking or pixelation, the low detection capacity of corner points is determined above a measurement distance of 30 m. Nevertheless, there are a couple of corner points detected in the bolt connections at 40 m; they are not functional because of a lack of the number of points, which is less than the minimum requirement of 3 points within each ROI for the MLESAC algorithm. The mean number of KAZE features within the bolt connection is extracted more than the minimum requirement for tracking at the longest measurement distance. Both feature detection schemes in slab cross-sections have limitations of feature detection starting from a measurement distance of 30 m.

In order to evaluate the vision sensor’s performance in the time domain, dynamic displacements of floors are estimated based on tracking key points extracted from both artificial and natural targets by implementing two feature detection schemes: the Shi-Tomasi corner detector and the KAZE feature detector. To simplify the expressions, the corresponding displacement measurements are denoted as STC and KZF, respectively. Additionally, they are compared to LVDT data in order to quantify measurement error using Root Mean Square Error (RMSE) and Mean Absolute Peak Error (MAPE).

**Figure 6:**
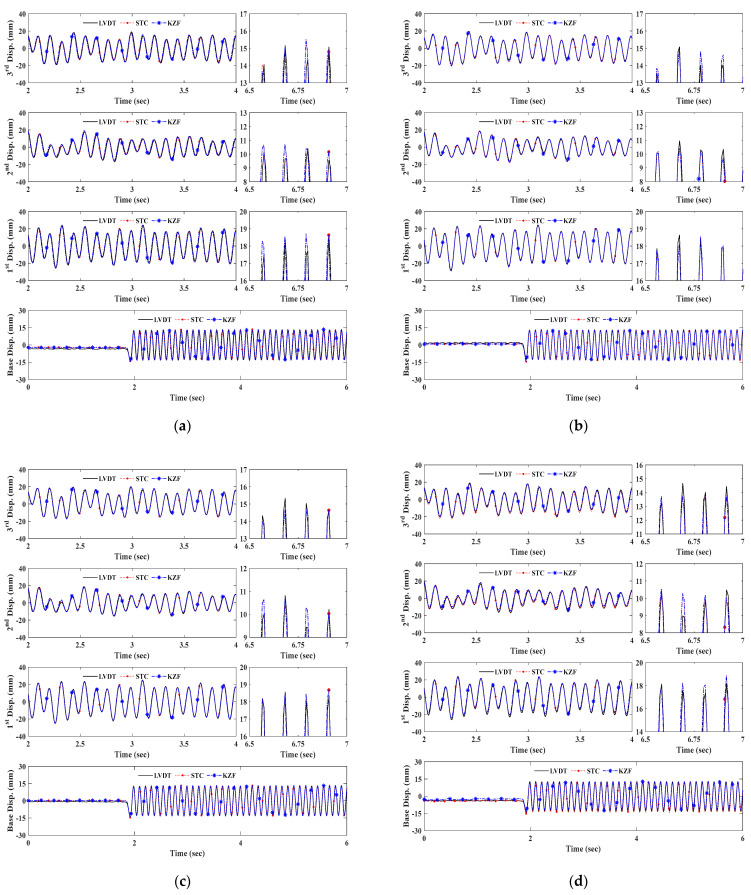
Displacement measurement comparison of artificial target. (**a**) Measurement distance of 10 m. (**b**) Measurement distance of 20 m. (**c**) Measurement distance of 30 m. (**d**) Measurement distance of 40 m.

Figure 6 shows the displacement time histories of the artificial target excited by 9 Hz frequency depending upon the measurement range increments under normal indoor illumination. The 1st, 2nd, and 3rd floors’ displacements are relative to the base table’s displacement. The relative displacement time histories of each floor are indicated in a duration of 2 s, and the enlarged plots are presented to provide better illustrations.

As presented in all the results, the feature point-based measurement system using artificial targets can accurately determine a wide range of relative vibration amplitudes ranging from 0 to 30 mm. Moreover, the vision sensor demonstrates significant displacement measurement performances in a high excitation frequency.

**Figure 7:**
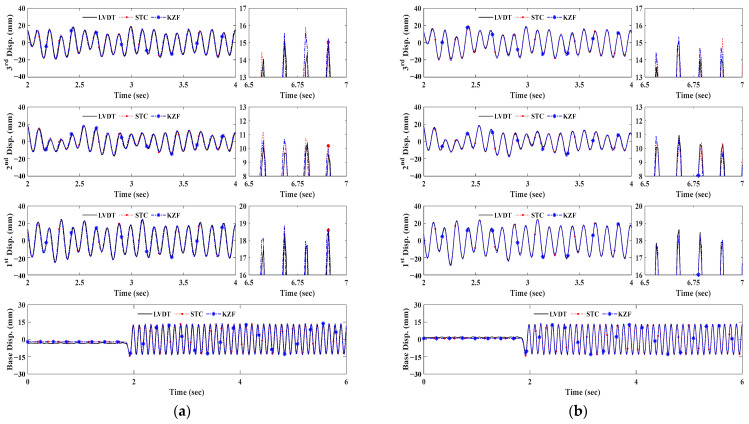

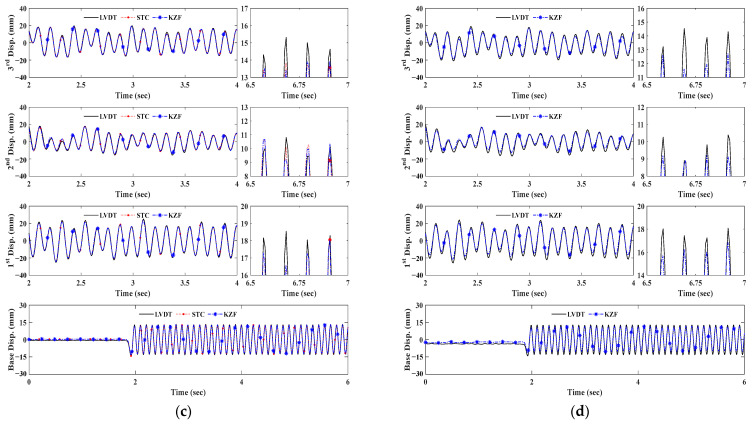
Displacement measurement comparison of bolt connection. (**a**) Measurement distance of 10 m. (**b**) Measurement distance of 20 m. (**c**) Measurement distance of 30 m. (**d**) Measurement distance of 40 m.

**Figure 8:**
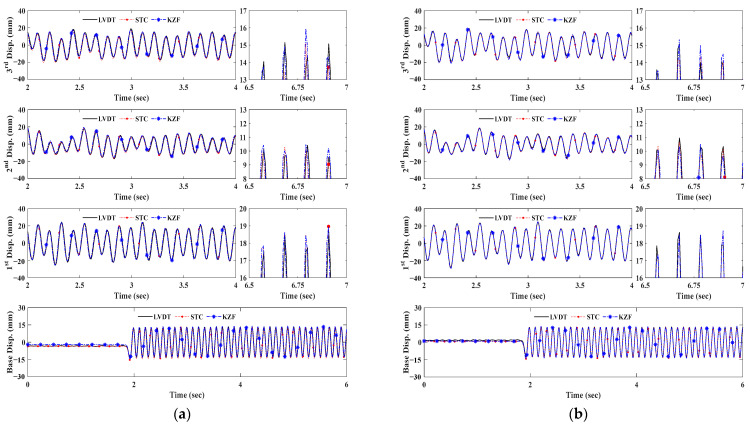
Displacement measurement comparison of slab cross-section. (**a**) Measurement distance of 10 m. (**b**) Measurement distance of 20 m.

Figure 7 and Figure 8 are the structural dynamic responses using two types of natural targets. As can be seen in the results at 10 m and 20 m, the STCs and KZFs show good agreement with the corresponding reference data. Because of the pixelation resulting from the increase in scale factor in the natural targets, the distance range limitation when using a slab cross-section was determined as 30 m in this testing configuration. Although both feature detection schemes are applicable in the bolt connection up to 30 m, the corner detector is unsuitable for tracking above 30 m due to insufficient features. Additionally, this would affect dramatic decreases in the measurement accuracy of bolt connections between 30 m and 40 m.

To estimate the displacement measurement performance as distance increases, error quantification was numerically studied, as tabulated in Table 2. The errors were quantified to RMSE and MAPE in mm and percentile, respectively. The RMSE of the fiducial target ranges from 0.61 mm to 0.74 mm in both feature detection schemes and shows a proportional relationship as measurement distance increases. The maximum MAPE of STC and KZF using fiducial target is 1.58% and 1.56%, respectively, at 40 m. As can be seen in the artificial target’s error quantifications, there is no big difference between the two feature detection techniques.

The RMSEs of STC and KZF slightly increase to 0.79 mm and 0.76 mm, respectively, at 30 m. However, there are sudden drops in the MAPEs of both displacement measurements at 30 m. Furthermore, the RMSE of KZF significantly increases to 1.2 mm and the corresponding MAPE sharply decreases to −10.2% at 40 m. Comparing the RMSEs and MAPEs using bolt connection, similar levels of measurement errors were determined in slab cross-sections at 10 m and 20 m.

Through comparative studies, the measurement distance is a critical factor in decreasing the measurement accuracy of a vision sensor. It can particularly be observed in small configurations of natural features because of the lower spatial resolution components as the measurement distance increases. Moreover, it is found that the KAZE features are more robust to measurement distance compared with corner points.

### 3.2. Feasibility Study of Super-Resolution in Vision Sensor-Based Measurement System

**Figure 9:**
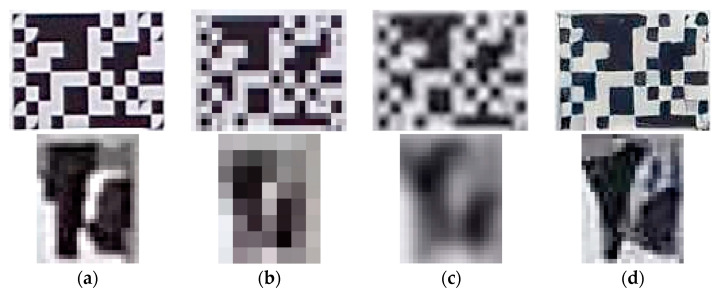
Visualization comparison of upscaled images. (**a**) Org. (1.0). (**b**) BC (0.25). (**c**) BC (1.0). (**d**) RealSR (1.0).

To validate the feasibility of SISR in a vision sensor-based measurement system, 2 sets of recorded image sequences of shaking table tests at 10 m and 20 m were used. All the original images were initially downsampled with a scale factor of 0.25 using BC operation, and the downscaled ones were separately enhanced with a scale factor of 4 through bicubic operation and RealSR. Figure 9 shows the artifact results of the artificial target and bolt connection on the frame structure at a measurement distance of 10 m. Compared with the original image (Figure 9a), the blurry images of two types of targets can be seen in BC images (Figure 9c). As shown in the fiducial target image (Figure 9d), the distinctive visual features were generated using RealSR.

Table 3 shows the quantitative analysis of the similarity of BC and SR images using 1000 corresponding reference image frames. Similar numerical results are shown in both SR operations at 10 m. However, improved SR performances are determined in both PSNR and SSIM of BC as the measurement distance increases. Because the PSNR and SSIM are normally applied for evaluation metrics in image restoration, they pay more attention to the fidelity of the image. Because RealSR uses perceptual loss for real-world SR images, it shows better SR performances in the LPIPS metric.

**Figure 10:**
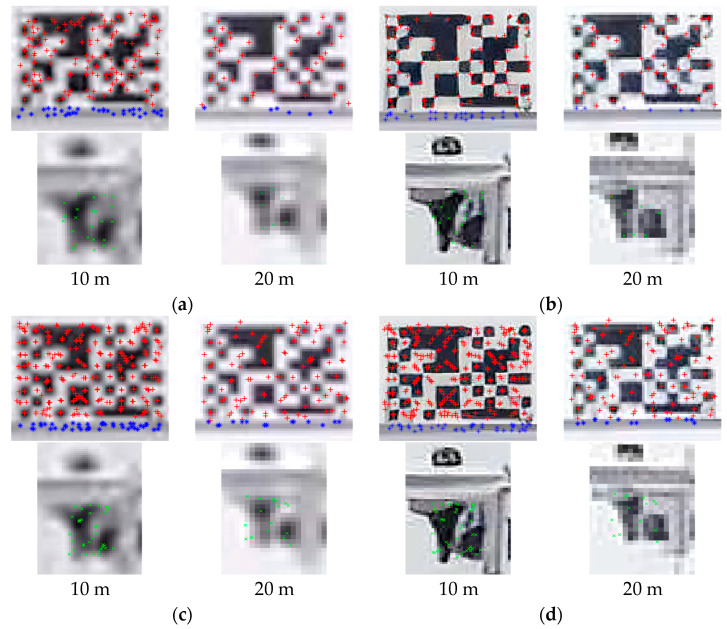
Corner point and KAZE feature detection results of bicubic upsampled and RealSR images at 10 m and 20 m. Red +, blue * and green × mean the detected points within the fiducial target, slab cross-section and bolt connection respectively. (**a**) Corner point detection (BC images). (**b**) Corner point detection (RSR images). (**c**) KAZE feature detection (BC images). (**d**) KAZE feature detection (RSR images).

The reconstructed image frames are applied to the feature point-based displacement measurement system. Figure 10 shows the feature detection results of artifacts generated by two SR methods. Due to the visual effects of RSR images, they provide distinctive features in terms of corner points, as shown in Figure 10b. Nevertheless, the KAZE feature detector shows secure detection performance, especially in blurry images (BC images) because of its non-linear diffusion filtering process.

Table 4 shows the error quantification of displacement measurements using upsampled images to evaluate the feasibility of SISR in the vision sensor-based measurement system. The RMSEs of displacement measurements using BC images are generally higher than those of RealSR. The standard deviations and mean of RMSEs depending upon BC and RealSR images are 0.06 and 0.05, and 0.68 mm and 0.67 mm, respectively. In the case of MAPEs, BC and RealSR images are 0.147 and 0.151, and 1.75% and 1.57% respectively. In terms of feature detection methods, the mean RMSE of KZF is 0.65 mm, which is 0.05 mm lower than its STC.

The smaller average RMSEs can be found in both displacement measurements using the artificial target in comparison with those using natural targets. Because of the high contrast of predesigned markers, distinctive features can be detected in both SR methods, leading to smaller RMSE differences compared to those using the natural targets. Comparing the two types of natural targets, the mean RMSEs of RSR images are smaller than those of BC images. Due to the realistic SR performance of RealSR, the SR model can contribute to enhancing the image quality of natural targets, i.e., bolt connection and slab cross-section. Thus, the SR model can be implemented for both improving image resolution and tracking motion trajectories in the feature-based measurement system.

### 3.3. Alleviation of Low Spatial Resolution Using Super-Resolution

The image sequences recorded in the previous experiments at 30 m and 40 m were used to alleviate low spatial resolution by applying SR images in the vision-based measurement system. In addition to the alleviation, the robustness evaluation was also carried out to investigate the measurement accuracy of the vision sensor depending on the increase in measurement distance. The original image frames were upscaled through the pre-trained model of RealSR with a factor of 2, and each enhanced image consisted of a 3840 × 2160-pixel image. Due to the increase in pixel density, the scale factors of artifacts at 30 m and 40 m decrease to 1.43 mm/p and 1.91 mm/p, respectively. The scale factor of the SR image at 40 m corresponds to the scale factor of the original image at 20 m.

**Figure 11:**
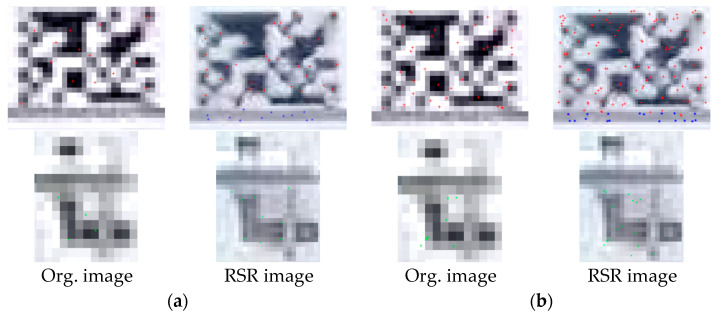
Corner point and KAZE feature detection results for RealSR images at 40 m. Red +, blue * and green × mean the detected points within the fiducial target, slab cross-section and bolt connection respectively. (**a**) Corner point detection. (**b**) KAZE feature detection.

Figure 11 shows the feature detection results at 40 m by comparing original image frames (left) and the higher resolution images (right). As observed in the fiducial target artifacts, the image quality was properly enhanced in indoor light conditions. The details of bolt connections in synthetics are lost owing to a lack of pixel information from the original ones. Despite ambiguities of natural targets in SR images, both points of interest were detected in all types of targets.

**Figure 12:**
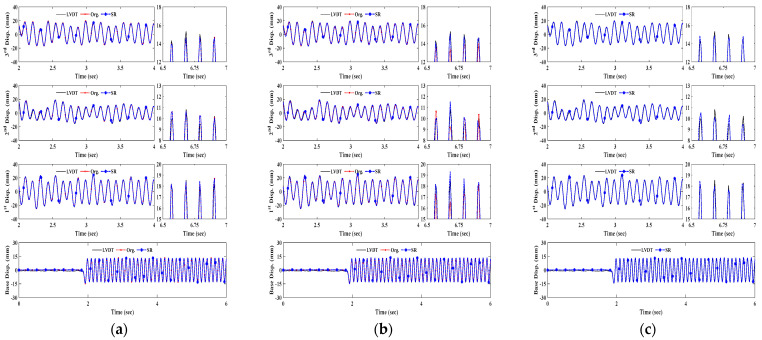
KZF comparison at 30 m depending on targets. (**a**) Artificial target. (**b**) Bolt connection. (**c**) Slab cross-section.

**Figure 13:**
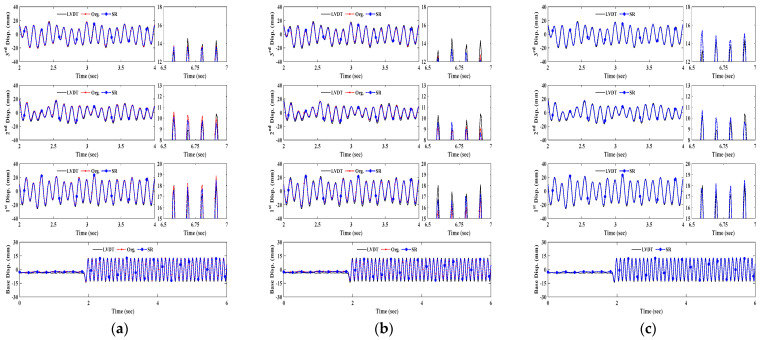
Displacement measurement comparison at 40 m depending on targets. (**a**) KZF using artificial target. (**b**) KZF using bolt connection. (**c**) KZF using slab cross-section.

Figure 12 and Figure 13 representatively plot displacement measurement time histories of LVDT data and KZFs using original and SR images depending on measurement distance. As can be seen in both figures for brief illustrations, the KZFs using SR image sequences of fiducial targets show great agreement with the reference data regardless of an increase in measurement distance range.

For the bolt connection, the discrepancies from the original images at 30 m and 40 m become smaller, as can be observed in the displacement comparisons, specifically in the enlarged time segments in both Figure 12b and Figure 13b. Owing to the increase in spatial resolution, the enhanced images of the slab cross-section allow tracking of the movement of each floor and extend the measurement distance range of the vision sensor (Figure 12c and Figure 13c).

Table 5 shows the error quantification of the robustness evaluation of SR in the feature point-based displacement measurement by refining the coarse details of image frames. All the RMSEs of displacement measurements using upscaled images of the artificial target and bolt connection become smaller compared to the corresponding measurements of the original images. The RMSEs of STC and KZF of the artificial target at 30 m decrease to 0.67 mm and 0.65 mm, respectively. Moreover, the corresponding MAPEs decrease to 1.44% and 1.42%, respectively. Similar to the case at 30 m, the higher spatial resolution caused a decrease in RMSEs and MAPEs as the measurement distance range was extended.

In the case of bolt connection, the RMSEs of STC and KZF at 30 m are 0.7 mm and 0.65 mm, respectively. Additionally, the absolute MAPEs of STC and KZF decrease to 1.96% and 1.80%, respectively. While the corner points can contribute to tracking the trajectory of the structure at 40 m because of higher pixel density, the RMSE and MAPE of STC at 40 m reached 0.77 mm and −5.10%, respectively. Similar to KZF at 30 m, the RMSE and MAPE of KZF at 40 m decrease to 0.87 mm and −4.84%.

For the slab cross-section, the SISR provides higher spatial resolution to detect key points, i.e., corner points and KAZE features within the ROIs at longer measurement distances. Compared to RMSE and MAPE of both measurements at 30 m using bolt connection, the improved measurement accuracy can be found in those of the slab cross-section. While similar measurement error trends can be observed in both STC and KZF at 30 m, the enhanced measurement accuracy can be determined in the KZF at 40 m by comparing the STC at 40 m. This is caused by the larger quantity of features and more distinctive points of interest in slab cross-sections that participated in tracking dynamic responses.

## 4. Conclusions

The dynamic responses of a frame structure were obtained by tracking trajectories of feature points within the predesigned targets and natural targets such as bolt connections and slab cross-sections. The fiducial targets provide distinctive ROIs to the Shi-Tomasi corner detector and the KAZE feature detector for extracting and tracking features at a measurement distance of 40 m. Through evaluation of the measurement performance of the feature point-based measurement system, excellent agreements can be observed between the displacements computed using the artificial targets and those measured by reference sensors. Unless natural targets are suitable for obtaining structural dynamic responses, the measurements using these targets have distance range limitations from 30 m. Additionally, their feature detection capacities are sensitive to the pixel blocking of images resulting from losing spatial density.

The GAN-based SISR was introduced in the vision sensor-based measurement system in order to alleviate the low spatial resolution due to the increase in measurement distance. The feasibility study of SISR in the vision-based sensor system was carried out to remotely measure multiple structural responses by tracking the natural features existing on structures at a long measurement distance. The pre-trained model of SR obtained outstanding SR results in terms of human perception by refining coarser image frames to high-resolution artifacts. Moreover, the SR images were functional to estimate displacement, and there are no big differences in the displacement measurement performances using reconstructed image frames compared with those of original images.

In addition to the feasibility study of SISR, the robustness of feature point-based displacement measurement in terms of extension of measurement distance was evaluated by increasing the spatial resolution of image sequences. Owing to the enhanced images, the measurement accuracy of the feature point-based measurement system was remarkably increased in the case of natural targets. Furthermore, the operational capacity of the vision sensor using natural targets was extended to 30 m in both feature detection schemes. While the displacements can be computed using the natural targets at 40 m, the KAZE features are more applicable for the lower contrast ROIs.

To sum up, it was found that the RealSR accomplishes refinement of definite images of natural targets, resulting in an extension of the measurement distance range in the feature point-based measurement system. Additionally, it is confirmed that the SISR mitigates displacement measurement error in the vision sensor-based measurement system at long measurement distance ranges. Based on this fundamental study of SISR in the feature point-based measurement system, further analysis such as modal analysis, damage detection, and so forth should be continued in order to explore the functionality of SR images by applying low-resolution displacement measurement footage.

## Figures and Tables

**Table 1:** Feature detection result comparison.

Feature Type	Target Type	Measurement Distance (m)			
		10	20	30	40
**Corner points (no.)**	A ^1^	90	82	41	29
	B ^2^	25	9	4	2
	S ^3^	46	9	-	-
**KAZE features (no.)**	A	288	100	54	34
	B	36	16	10	5
	S	55	16	-	-

^1^ Artificial target, ^2^ bolt connection, and ^3^ slab cross-section.

**Table 2:** Error quantification of vision-based measurement system depending on the increase in measurement distance.

Disp. Measurement	Error	Target Type	Measurement Distance (m)			
			10	20	30	40
**STC**	**RMSE (mm)**	**A ^1^**	0.62	0.67	0.70	0.75
		**B ^2^**	0.65	0.69	0.79	-
		**S ^3^**	0.66	0.67	-	-
	**MAPE (%)**	**A**	1.38	1.45	1.52	1.58
		**B**	1.74	1.85	−4.30	-
		**S**	1.55	1.57	-	-
**KZF**	**RMSE (mm)**	**A**	0.61	0.63	0.66	0.74
		**B**	0.63	0.65	0.76	1.20
		**S**	0.66	0.68	-	-
	**MAPE (%)**	**A**	1.40	1.43	1.50	1.56
		**B**	1.72	1.81	−4.19	−10.22
		**S**	1.56	1.59	-	-

^1^ Artificial target, ^2^ bolt connection, and ^3^ slab cross-section.

**Table 3:** Quantitative result of image restoration.

Measurement Dist. (m)	PSNR (dB) ↑		SSIM ↑		LPIPS ↓	
	BC ^1^	RSR ^2^	BC	RSR	BC	RSR
10	32.11	32.34	0.91	0.90	0.29	0.21
20	35.85	32.92	0.95	0.87	0.22	0.22

↑ and ↓ mean higher value or lower value is desired. ^1^ Bicubic upsampling and ^2^ RealSR.

**Table 4:** Error quantitative analysis.

Disp. Measurement	Error	Target Type	Measurement Distance (m)					
			10			20		
			Org.	BC ^4^	RSR ^5^	Org.	BC	RSR
**STC**	**RMSE (mm)**	**A ^1^**	0.62	0.72	0.70	0.67	0.68	0.64
		**B ^2^**	0.65	0.76	0.73	0.69	0.72	0.68
		**S ^3^**	0.66	0.74	0.70	0.67	0.70	0.65
	**MAPE (%)**	**A**	1.38	1.59	1.43	1.45	1.53	1.37
		**B**	1.74	1.84	1.68	1.85	1.95	1.80
		**S**	1.55	1.79	1.63	1.57	1.77	1.61
**KZF**	**RMSE (mm)**	**A**	0.61	0.69	0.69	0.63	0.59	0.59
		**B**	0.63	0.69	0.70	0.65	0.59	0.59
		**S**	0.66	0.70	0.71	0.68	0.60	0.60
	**MAPE (%)**	**A**	1.43	1.61	1.44	1.43	1.63	1.47
		**B**	1.72	1.87	1.71	1.81	1.99	1.84
		**S**	1.55	1.78	1.62	1.59	1.66	1.50

^1^ Artificial target, ^2^ bolt connection, ^3^ slab cross-section, ^4^ bicubic upsampling, and ^5^ RealSR.

**Table 5:** Error quantification of vision-based measurement system using upscaled images.

Disp. Measurement	Error	Target Type	Measurement Distance (m)			
			30		40	
			Org.	RSR ^4^	Org.	RSR
**STC**	**RMSE (mm)**	A ^1^	0.70	0.67	0.75	0.72
		B ^2^	0.79	0.70	-	0.77
		S ^3^	-	0.68	-	0.85
	**MAPE (%)**	A	1.52	1.44	1.58	1.54
		B	−4.30	1.96	-	−5.10
		S	-	1.74	-	−6.80
**KZF**	**RMSE (mm)**	A	0.66	0.65	0.74	0.68
		B	0.76	0.65	1.20	0.87
		S	-	0.63	-	0.76
	**MAPE (%)**	A	1.50	1.42	1.56	1.45
		B	−4.19	1.80	−10.22	−4.84
		S	-	1.58	-	1.61

^1^ Artificial target, ^2^ bolt connection, ^3^ slab cross-section, and ^4^ RealSR.

## Data Availability

Not applicable.
